# Is it not too redundant? When signaling overlap reduces extraneous load and enhances retention in a software video tutorial

**DOI:** 10.3389/fpsyg.2026.1795142

**Published:** 2026-05-22

**Authors:** Juliette C. Désiron, Tino Endres, Sascha Schneider

**Affiliations:** 1 Department of Teacher Education, University of Fribourg, Fribourg, Switzerland; 2 Institute of Education, Educational Technology, University of Zürich, Zurich, Switzerland; 3 Institut für Psychologie, Albert-Ludwigs-Universität Freiburg, Freiburg, Germany; 3 Faculty of Psychology, UniDistance Suisse, Brig, Switzerland

**Keywords:** cognitive load, multimedia learning, redundancy, signaling, video tutorial

## Abstract

The redundancy effect in Cognitive Load Theory posits that presenting overlapping information across multiple channels (e.g., duplicate auditory and visual content) can impede learning by increasing extraneous cognitive load and hindering mental model integration. In this study, we tested a boundary condition for this effect in the context of software video tutorials, in which redundant information takes the form of spatially integrated signaling with task-relevant interface elements. Specifically, we examined whether the *contentual* redundancy effect persists as of visual signaling increases in a complex software training context, or whether such signal redundancy instead supports learning. Using a between-subjects design, we systematically increased the amount of visual signaling in a Photoshop tutorial video across five groups. All groups received identical auditory guidance but differed in the amount of visual signaling provided. Linear contrast analyses showed that higher visual signaling was associated with higher retention and transfer performance, as well as reduced extraneous load. Interestingly, higher amounts of signaling increased germane cognitive load and the mental effort invested. These results indicate that, in complex software tutorials, content redundancy introduced through spatially integrated signaling does not hinder learning; instead, it can reduce extraneous load and foster germane processing.

## 1 Introduction

Mastering software today often starts with a video tutorial. Whether for professional tools or everyday applications, learners increasingly turn to platforms such as YouTube and MOOCs for quick, practical guidance. This trend reflects a broader shift: video tutorials offer an accessible, visual, and step-by-step approach that simplifies complex interfaces and workflows. Unlike static manuals, they allow learners to see processes unfold in real time, making abstract commands concrete and actionable. As digital skills become essential across fields, the ability to learn software efficiently through video is not just convenient—it is critical for keeping pace with technological change.

This growing reliance on video tutorials aligns with broader developments in online education. E-learning, online-based or fully online learning, is becoming an increasingly important part of the available learning content (Bach, 2019). As online education continues to grow and gain traction (Allen and Seaman, 2017), so does the use of video (Guo et al., 2014; Ragazou and Karasavvidis, 2021b). Instructional videos are not only common in formal classrooms but also play a central role in informal learning settings (Merkt et al., 2018). Platforms such as YouTube and MOOCs largely contributed to the surge in video production, which serves to demonstrate and explain how to acquire the necessary knowledge or skills to master complex topics or environments in a compact, structured, and vivid manner (de Koning et al., 2018). Recent evidence from authentic instructional videos further suggests that multimedia principles are implemented unevenly in practice, with signaling present in many but not all videos, underscoring the need to examine how signaling is used and how beneficial it is in real instructional contexts (La Torre and Désiron, 2024).

### 1.1 Learning with and from video

Videos have been integrated into teaching materials since the early 1900s, with motion pictures produced as short visual supports for instructional lessons (Mayer et al., 2020). Video production gradually evolved with the growth of educational videos (see Cohen, 2014), which targeted specific educational needs rather than the broader audience of motion pictures. Another shift toward the public was observed in the 2000s (van der Meij and van der Meij, 2015), marked by the emergence of online tutorials (e.g., how to braid one’s hair; simple Excel features) and recorded public presentations (e.g., TED Talks). Finally, in recent years, the development of MOOCs has initiated a new wave of educational videos (Guo et al., 2014). Whether targeting a large audience or a specific group of learners, these video types also differ in their representational formats. Instructional videos can be live-action (e.g., enactment of a historical event), animations (e.g., Once upon a time… Life series, computer-generated animation of the piano mechanism), blackboard demonstration [e.g., Math lesson on Khan Academy, 2006], recorded lesson with slides and narration [e.g., statistic lessons by Field, 2012, on YouTube], or video examples (e.g., tutorial on knot tying).

While the integration and format of instructional videos are closely linked to technological evolution, from a research perspective, they can be theoretically grounded in the cognitive theory of multimedia learning (CTML; Mayer, 2021). Briefly, this theory describes the processing of multimedia information during learning and advocates presenting visual information with narration (*modality principle*) to support dual encoding (Baddeley, 1992; Paivio, 2006), while limiting cognitive overload (Baddeley, 1992; Sweller et al., 2011a). However, merely using multiple representations is insufficient to foster learning; careful design choices that guide learners’ attention can be beneficial (e.g., de Koning et al., 2010; Fischer and Schwan, 2010). Mayer (2021) thus reports a series of design *principles—*also referred to as *effects* in research dedicated to cognitive load*—*that should support the integration process and decrease cognitive load: (1) minimize extraneous processing, (2) manage essential processing, (3) foster generative processing. However, the CTML is a general framework for multimedia learning, and not all principles apply to the design of instructional videos, and all come with boundary conditions which includes learners’ characteristics, kind of lesson and learning context (Cromley and Chen, 2025; Mayer, 2024).

Several empirical studies have attempted to bridge this gap by systematically reviewing the literature on instructional videos and reporting on applicable design principles. In their exploratory study, Fyfield et al. (2019) built on Mayer’s design principles for multimedia documents and proposed 25 principles for instructional video design. These principles were derived from empirical research based on a systematic review of the literature, including both experimental and qualitative studies. In addition to the three design principles’ categories from the CTML (Mayer, 2021), the authors included a category for principles dedicated to interface design. Furthermore, some of the principles retained from the CTML were subdivided to provide more precise design guidelines (e.g., a distinction between visual and auditory seductive details). The guidelines are listed and illustrated but observed or expected effects on learning outcomes or cognitive load are not clearly indicated. The systematic review by Castro-Alonso et al. (2021) focused on five design strategies aimed at reducing either learners’ or instructors’ cognitive load generated by material problems. It addressed both cognitive load theory (CLT, as an effect) and CTML (as a principle). These five strategies are developed for all types of instructional multimedia, and two apply to video design: (1) signal essential information and (2) segment dynamic information (system or user-pacing). Furthermore, the researchers point out that novice or struggling learners benefit most from implementing these strategies directly in the design or by instructors, whereas those with greater expertise benefit most from self-regulation.

### 1.2 Signaling to support learning

To aid learning with video tutorials, classical literature on multimedia learning suggests including signaling cues (Castro-Alonso et al., 2021; Noetel et al., 2022). Mayer describes the signaling principle as “[h]ighlight[ing] essential material” (Mayer, 2021, p. 69). This definition was further elaborated by de Koning et al. (2009), who distinguished three functions of signals: attention guidance, emphasis, and salience. In multimedia documents, signals facilitate learners’ linking of media by highlighting redundancy between visual and verbal information. While the *redundancy* principle in multimedia learning (Kalyuga and Sweller, 2021) emphasizes that redundant information likely to increases cognitive load, signaling this redundancy can help learners (Désiron et al., 2021b; van Gog, 2021). Research has shown that signaling effectively reduces learning time (Mayer, 2021) and cognitive load (Schneider et al., 2018).

Learning outcomes in multimedia research on the signaling effect typically encompass retention, defined as the recall of elements clearly stated in the material, and transfer, defined as the ability to reuse acquired knowledge in a similar situation (Mayer, 2021). The latter is particularly suitable for problem-solving or procedural instructional material. Some research has measured comprehension, which is often contrasted with retention, as it requires learners to generate inferences within the document or using prior knowledge when constructing their mental model of the situation (Désiron et al., 2021a).

Cognitive load theory (CLT) relies on principles regarding information processing and storage. In multimedia research (Klepsch and Seufert, 2020; Mayer, 2021), the term is used to describe limitations in working memory processing, including the amount of information and the retention time. Three types of cognitive load are usually distinguished: *Intrinsic—*triggered by the learning content (ICL), *extraneous—*depending on the design (ECL), and *germane—*corresponding to available working memory capacity (GCL). Ideally, design properties that support learning, such as signaling that decreases ECL, should be implemented to provide more processing power devoted to ICL and, in turn, prevent GCL.

Learning processes can also be considered more broadly and include metacognitive monitoring as well as motivation experiences such as situational interest. For metacognitive monitoring (i.e., judgments of learning), according to cue-utilization accounts, are based not only on actual knowledge but also on the cues available at the time of judgment and their perceived diagnosticity (Koriat, 1997). One such cue may be the cognitive load experienced during learning (de Bruin et al., 2020). Research on cognitive skill acquisition further suggests that self-assessment accuracy depends on the nature of cues learners rely on, such as performance-based or explanation-based cues, and that cue use can be improved through instructional support (Waldeyer et al., 2024). In software tutorials, signaling may therefore affect metacognitive monitoring by making relevant task states and solution steps more salient and easier to evaluate when learners form judgments of learning. With regard to motivation, situational interest can be triggered by learners experience with the learning materials (Endres et al., 2020; Endres et al., 2025b).

#### 1.2.1 The effectiveness of signaling

Signaling in instructional multimedia has been studied a lot with written text and pictures (Désiron et al., 2021a; Kalyuga et al., 1999; Lehmann and Seufert, 2020; Richter et al., 2018; Scheiter and Eitel, 2015), as well as with instructional video in the animation format (Amadieu et al., 2011; Boucheix et al., 2013; de Koning et al., 2010, 2011; Désiron et al., 2021b). Based on this extensive research, van Gog (2021) defined signals as a means of highlighting corresponding verbal and visual representations implemented in the text representation, the visual representation, or both. Several meta-analyses have examined the signaling principle in multimedia documents (Alpizar et al., 2020; Richter et al., 2016; Schneider et al., 2018; Xie et al., 2016, 2017). An overall beneficial signaling effect for instructional multimedia was found on measures of learning outcome, along with a moderating effect of prior knowledge*—*in line with the *expertise-reversal effect* (Kalyuga, 2021; Sweller et al., 2003). A positive effect on cognitive load was reported as well. The meta-analysis from Xie et al. (2017) included a meta-regression to assess whether reductions in perceived cognitive load from signaling lead to better retention or transfer. The meta-regressions were consistent with the CLT: the effect of signaling on cognitive load significantly predicted signaling’s on both types of learning outcomes. In other words, when signaling decreases cognitive load, it increases learning outcomes. Noetel et al. (2022) conducted a meta-meta-analysis of several multimedia design principles to support learning and reduce cognitive load. For signaling, the five meta-analyses cited above were included, and an overall small, pooled effect size (*g* < 0.2).

#### 1.2.2 A wide variety of signals

Nevertheless, only a few studies have investigated signaling in instructional videos, and most have focused on animated format. Amadieu et al. (2011) implemented signaling by zooming in on key steps in an animation of the long-term potentiation process. Signaling did not affect cognitive load (mental effort and perceived difficulty) or retention. However, there was an interaction between signaling and exposure times on comprehension, with higher comprehension scores for signaled material. Boucheix et al. (2013) compared different signaling formats on learning about the piano key mechanism: (1) transparent coloured animation, (2) progressive arrows appearance, and (3) localised coordinated colours. Results confirmed that signaling led to higher comprehension of the material, although there was no difference between signaling conditions. Both studies, however, were used without the support of a second medium, either text or narration. Désiron et al. (2021b) studied cross-representational signaling in short looping animations presented in contiguity with written text, compared with no signaling and a text-only control condition. In this research, written text was used rather than narration, as the animation supported text comprehension. They found a positive effect of signaling in text with animation compared with the text-only, but not compared with the multimedia condition, and only for comprehension of global inferences. Overall, these studies infringe the modality principle, and there are yet no studies that use narrated animations. However, some research investigates signaling in video examples, most of which include audio narration.

### 1.3 Signaling in video examples

While there are studies on the effects of signaling in animations, research on signaling in filmed instructional videos remains scarce. Indeed, recent research on these instructional video formats often focuses on effects associated with the instructor (e.g., on-screen presence, voice tone, and attitude) or with production features (e.g., camera viewpoint). Some research (Expósito et al., 2020; Ibrahim et al., 2012) examined the implementation of several multimedia design principles together (e.g., segmenting, signaling) and found higher learning outcomes when following the CTML design principles. One of the few studies on signaling in filmed instructional videos was conducted by Jamet and Fernandez (2016), which examined a tutorial on using a web service (i.e., a video example). They found a positive effect of signaling on retention but no significant effect on transfer (procedural task) or on cognitive load. In addition to this study, Ragazou and Karasavvidis (2021a,b) examined the effects of signaling in software tutorial videos on task performance (declarative and procedural knowledge and transfer), mental effort, and motivation. They found no main effects or interaction effects of the signaling and practice manipulations on performance or mental effort. A major limitation of this study lies in its design: participants were all experts in the domain, and it has been repeatedly found that domain knowledge can affect the benefit of signals (Richter et al., 2016; Alpizar et al., 2020). In multimedia research, the effect of prior knowledge has been studied in the context of the expertise-reversal effect (Kalyuga, 2021), which suggests that implementing signaling and other supporting design principles may benefit novices but be detrimental to experts (Richter and Scheiter, 2019; Spanjers et al., 2011). Finally, Rodemer et al. (2021) used video examples in the form of case comparisons of similar chemical reactions, presented as equations, with an audio description of the depicted cause-and-effect. Signaling was tested using static color coding of equation components or the simultaneous presence of a semi-transparent, dynamic red dot on the part in question. Results showed that dynamic signals were more beneficial for learning outcomes than static signals or no signals.

A series of studies approached the question of signaling in video examples from the perspective of Eye Movement Modeling Example (EMME; for a review, see de Koning and Jarodzka, 2017). EMME is used as a signaling device in video examples to teach learners how to process multimedia material. The goal of this type of signaling was to guide learners’ attention to support the integration of multiple representations, rather than to direct their attention to relevant or linked information within those representations (e.g., swimming fish; Jarodzka et al., 2013). Overall, studies using EMME found that this signaling device improved processing of subsequent multimedia documents; however, effects on learning outcomes varied (Jarodzka et al., 2013; Krebs et al., 2019; Scheiter et al., 2018). Furthermore, consistent with Scheiter et al.'s (2018) assumption, Wang et al. (2020) found that EMME guided visual processing of the material rather than cognitive processing of the information and thus did not trigger an expertise-reversal effect.

This research on signaling in video examples that guide visual or cognitive processes provides an initial glimpse into the presence and design (e.g., static vs. dynamic) of signals that affect learning and/or cognitive load. Nevertheless, it remains unclear if such visual aids help learners with basic content prior knowledge and technical skills. Indeed, for online video tutorials, such as software tutorials, the target learners are often those with some prior experience (van der Meij and van der Meij, 2015).

### 1.4 Signals’ implementation as a moderator of the signaling effect

Although previous research shows that signals can be detrimental for learners with high prior knowledge of the content to be learned, little is known about learners with intermediate prior knowledge. Although the types of signals have been characterized in previous research (de Koning et al., 2009; Désiron et al., 2018; Richter et al., 2016; van Gog, 2021), the amount of signaling and the extent of their *contentual* redundancy remain open questions.

#### 1.4.1 How much signaling is needed to support learning?

The study by Lehmann and Seufert (2020), which used written text and static pictures, is among the few to consider the extent of signaling alongside measures of learning outcomes and cognitive load. Results of the study, comparing 4 levels of signaling (none to high), showed that low- to medium-level signaling was more beneficial for retention and transfer performance and was also associated with a lower ECL load than no or high signaling. No significant differences were observed in comprehension, GCL, or ICL. However, these results may be confounded by the quality of the signaling design, which increases with the extent of highlighting (from merely bold to brightly highlighted text sections). The number of signals has also been studied in a few studies using instructional videos in an animated format [for a review, see for example, de Koning et al., 2010 investigated signaling with single versus multiple elements simultaneously]. Xie et al. (2019) addressed the question of the amount of signals by means of signals’ modality, and Arslan-Ari et al. (2020) through signals’ localization. De Koning et al. (2010) studied signaling and animation—without text or narration—through spotlights, where all but the signaled elements are shaded. Learning outcome—including retention, comprehension, and transfer questions—was not affected by the amount or presence of signals. Also, no significant effect on cognitive load—assessed as mental effort—was detected. A comparison of signal modality (visual, audio, or both) and coordination (sequential or simultaneous) in a narrated animation by Xie et al. (2019) showed higher learning outcomes with dual-modality signaling and simultaneous presentation. Results from Arslan-Ari et al. (2020) on the localization of colour cues in a narrated animation with labels showed no effect of signaling, nor of the amount of signaling, on learning outcomes (retention, transfer, and matching task) or on cognitive load. Nevertheless, these studies used instructional animations, a specific type of instructional video that was particularly developed in the 2000s (Mayer et al., 2020). Furthermore, this prior research examined the extent of signaling by type or location rather than the total number of signals, as Lehmann and Seufert (2020) did with static pictures and written text.

#### 1.4.2 The fine line between supporting guidance and redundant guidance

Following the CLT, it is preferable to present the same information across two channels rather than in a single channel to avoid overloading any single channel (Kalyuga and Sweller, 2021). A typical example is to avoid overloading the verbal channel by presenting text in both written and audio formats simultaneously. However, according to Sweller et al. (2011b), p.141, “[a] redundancy effect may occur when the multiple sources of information can be understood separately without the need for mental integration.” In such cases of redundancy, the learning material is assumed to increase ECL, as learners unnecessarily process redundant information to integrate it with essential information. To sum up, presenting information in two channels should decrease load, but a redundancy effect occurs when learning is lower with the redundant information than without it.

The CLT also proposes a more fine-grained definition of the redundancy effect by distinguishing between *contentual* and *contextual* redundancy (Albers et al., 2023). The *contentual* redundancy corresponds to the definition of the redundancy principle in CTML and focuses on the overlap of information between representations (i.e., at the stimulus level). The *modal—or contextual—*redundancy refers to information that is duplicated between modes (i.e., at the sensory level). *Modal* redundancy does not necessarily assume *contentual* redundancy. For example, if the duplicate content is presented in both written text and an image, both *modal and contentual* redundancy effects occur. However, when different or complementary information is presented in the written text and the picture, it corresponds only to *modal* redundancy. Although redundancy typically arises when information is presented across different modalities, the CLT framework holds that any information that is unnecessary for learning is redundant. Although there are, to date, few studies investigating contentual redundancy in multimedia documents, the review by Trypke et al. (2023) reports substantial heterogeneity across studies, both in the implementation of redundancy (visual, auditory, or both) and in the reported results. The main reported moderating factor was the degree of interactivity between the representations and the redundant element. Furthermore, the *contentual* redundancy was consistently implemented through verbal and written elements (sentences, labels).

To sum up, redundancy is not merely the presence of multiple intelligible sources; it can be operationalized through two distinct lenses: contentual redundancy, which involves the contentual overlap of information across stimuli, and modal redundancy, which involves the simultaneous demand on a single sensory channel. While the expertise reversal effect is often explained by the redundancy principle, wherein information useful to novices becomes redundant for experts, the redundancy principle itself applies broadly to any information unnecessary for learning, regardless of the learner’s expertise level (Kalyuga, 2021).

## 2 Research aim

With the increasing use of technology, learning opportunities have expanded and are particularly facilitated in informal contexts. One example is the production and widespread diffusion of instructional videos on institutional platforms (MOOCs) and in public spaces (YouTube, Vimeo, etc.). In an informal setting, tutorial videos provide a guided explanation of new technologies or software functions. One particularity of these materials is that they are not intended for low-knowledge learners; instead, basic knowledge and skills are assumed.

To support video processing and facilitate learning with video tutorials, classical literature on multimedia learning suggests including signals. However, while signaling has been found to benefit low-prior-knowledge learners, it is unclear whether such aids benefit or harm learners with some prior knowledge, or how much signaling is needed.

The present study focuses on the extent of nonverbal signaling in instructional video examples. We operationalized the amount of signaling in the tutorial video as the *contentual* redundancy between signals and the audio description, rather than as a gradual increase in signaling. Our goal was thus to determine the degree of signaling redundancy required to guide learners’ attention, thereby reducing cognitive load and improving learning from a tutorial video. Indeed, consistent with CTML and prior research, highlighting core elements in the material by inserting cues should reduce processing of extraneous information and foster learning.

We expected retention and transfer to increase significantly as signaling increases (Learning outcome hypothesis, H1). As signals constitute additional attentional guidance elements, they should decrease ECL (Extraneous load reduction hypothesis, H2) while increasing GCL (Germane load increase hypothesis, H3). In addition, we explored the effect of signaling on other learning processes, namely metacognition and situational interest. Finally, mental load and mental effort were assessed to examine whether the signaling effects were mirrored in broader ratings of cognitive demand and invested effort during memory retrieval. We tested the same hypotheses exploratively on those measures.

## 3 Methods

### 3.1 Design and participants

A one-factorial between-subject design was used to test the research hypotheses. Based on the mean effect size of the meta-analysis of Schneider et al. (2018), *η_p_*^2^ = 0.06 *R*^2^ = 0.13, a power analysis was carried out with G*Power (Faul et al., 2007) with a test power of 1 – *β* = 0.80, and an error probability of *α* = 0.05. To detect the linear trend of our hypotheses a minimum sample size of *N* = 59 participants is required.

One hundred and one students from a German university (76.2% female, 22.8% male, 1% diverse; M_age_ = 23.84 years, SD = 3.14) participated in the study. After answering prior knowledge and demographic questions, they were randomly assigned to one of five signal redundancy conditions: Basic Level (*n* = 20), Position Frame (*n* = 20), Highlighted Frame (*n* = 20), Workspace Frame (*n* = 20), Macro Frame (*n* = 21).

### 3.2 Learning material

The learning material was designed as a software tutorial video about functions in Adobe Photoshop. This was chosen because of the program’s high complexity and restricted access. The goal was to provide primarily content for learners already familiar with the software who seek to learn more advanced functions and settings. Thus, the content covered specific tools, functions, and settings. The instructional video started with a brief reminder of the program layout and main icons, to follow the pre-training principle and ensure that all basic elements were known to learners. Then, the tutorial video focused on the differentiation of layer types. For smart objects and pixel layers, the advantages and disadvantages were demonstrated and explained. In addition, the contrast settings were presented by showing how changes in curves affect the image and by explaining the meaning of RGB values in the colour space. Finally, various approaches to creating object selections were discussed. The tutorial video consisted of a screen recording with a voice-over. The narration was delivered in a non-digital female voice, in accordance with the voice principle recommendations.

The videos produced for the experimental condition were presented online. The website was configured to display no video controls, preventing changes to viewing time or speed and thereby improving the standardization of experimental conditions. As a result, the learning time could not be influenced by the users. The video duration was 22:52 min across all experimental conditions.

To enable a clean comparison of the experimental groups, a baseline video was created and used as the basis for each signaling manipulation.

### 3.3 Experimental manipulation of signaling redundancy

**Figure 1:**
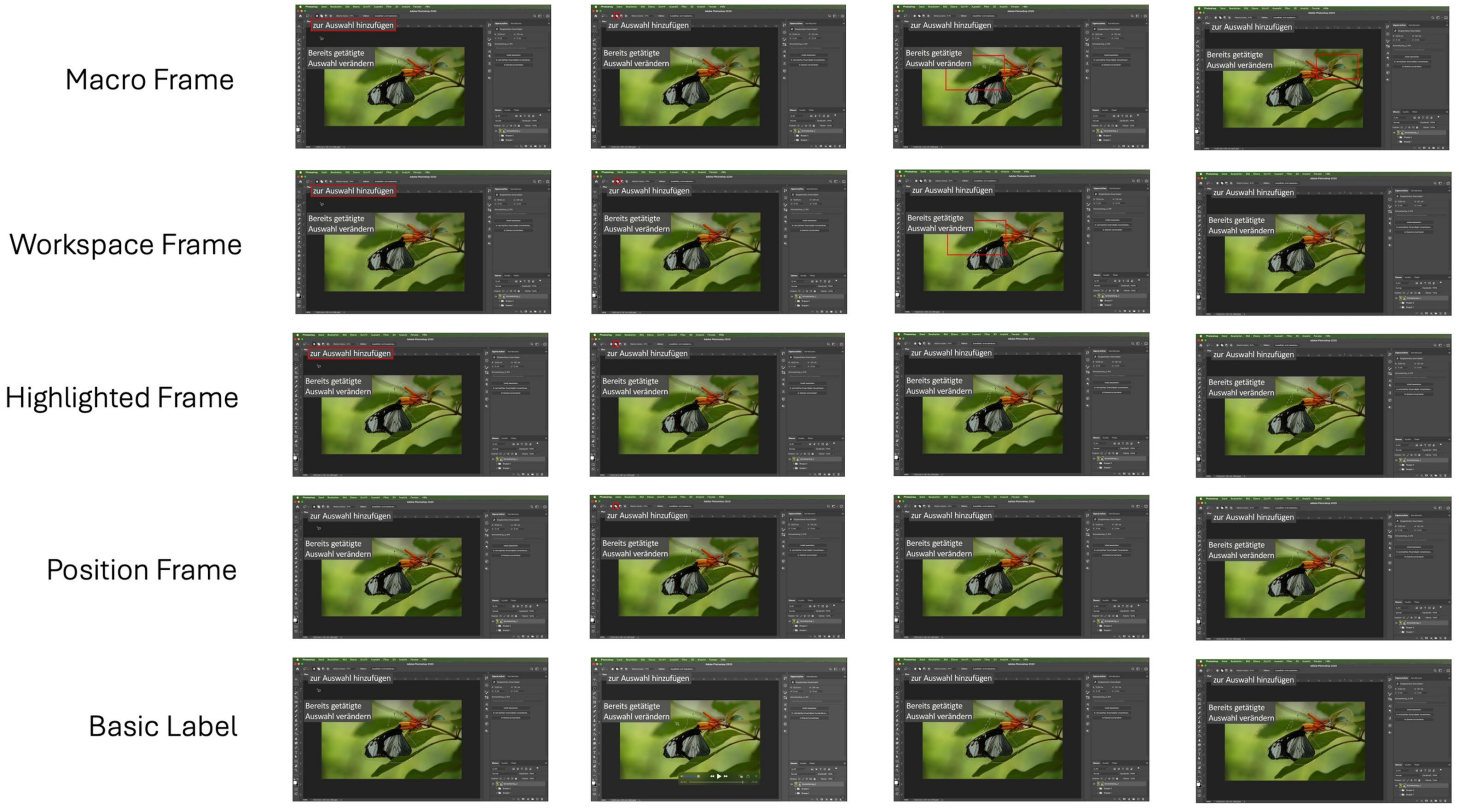
Screenshots of the software video tutorial illustrating the five conditions (timestamps: 20:41–20:46).

Based on the literature, signals were gradually integrated into instructional videos to support novices using Adobe Photoshop, linking visual and verbal information by highlighting where to focus their visual attention. All videos included identical auditory guidance but differed in how much of the provided signals were contentually redundant. In the Basic Label version, only visual labels of tool names were displayed; in the Position Frame version, the position of tools in the toolbar was highlighted using a red frame; in the Highlighted Frame version, a secondary frame was incorporated around the tool labels; in the Workspace Frame version, a frame was added around the workspace, when it was the current area of interest; in the Macro Frame version, framing of additional specific area of interest in the workspace were added. All visual signals were displayed when the corresponding elements were mentioned in the audio commentary, to comply with the *temporal contiguity* principle of the CTML (Fiorella and Mayer, 2021). In other words, the degree of contentual redundancy (i.e., the extent of overlap between visual cues and the audio commentary) gradually increased between conditions, as each additional signal (label and frames) provided a visual repetition of information concurrently mentioned in the audio track. Screenshots from 20:41 to 20:46 illustrating the five conditions are presented in Figure 1.

### 3.4 Measures

#### 3.4.1 Prior knowledge

The participants’ prior knowledge was assessed using four open-ended questions about their experience with image processing in general, their experience with the program Photoshop specifically, and their intended personal use. This resulting value thus represents the participants’ self-assessment of their prior knowledge. The open answers to the intended use and the description of use were rated by two independent raters on a scale from 0 to 3, analogous to beginner to professional [*κ* = (0.761; 766)]. Prior knowledge score (*α* = 0.790) was computed with a sum (min. 0, max. 12). Software expertise (*α* = 0.709) was measured with a short multiple-choice test with questions on general application basics (four questions, five multiple-choice answers) and the assignment of functions to icons (five questions, five multiple-choice answers).

#### 3.4.2 Learning outcome

Measures of learning outcome targeted retention and transfer. Retention was assessed using percent correct across 21 single- and multiple-choice questions (*α* = 0.717). The questions related to the recognition of icons and the correct assignment of functional possibilities, as well as questions about the range of functions of tools and tool groups. In addition, differences between the level types were examined, including their possible uses and their advantages and disadvantages. Transfer was assessed using percent correct across eight sorting, multiple-choice, and open-ended questions (*α* = 0.668). Participants had to pick the correct description for a specific software parameter or function.

#### 3.4.3 Cognitive load

Measures of ECL (3 items, *α* = 0.89), ICL (2 items, *α* = 0.88), and GCL (3 items, *α* = 0.70) were items adapted from the scale developed by Klepsch et al. (2017) and presented with a 9-point Likert scale.

#### 3.4.4 Variables for exploratory analysis

##### 3.4.4.1 Cognitive demand in answering

To assess cognitive demand while answering the questions, we distinguished between intentionally invested mental effort and perceived mental load, following the MEL-TS framework (Endres et al., 2025a). We assessed mental effort (6 items; Cronbach’s *α* = 0.842) and mental load (6 items; Cronbach’s *α* = 0.876, Krell, 2015).

##### 3.4.4.2 Situational interest

We assessed situational interest using a single item adapted from Endres et al. (2020), “How interested were you in the content shown in the video?” After the activity, learners rated their current interest in the topic on a 1–9 scale (1 = not at all, 9 = very much). The rating scale was used for the score.

##### 3.4.4.3 Judgement of learning

To assess learners’ metacognitive monitoring, we assessed the judgment of learning (JOL). We requested learners to rate their ability to answer questions such as those posed in the pretest with a single item: “On a scale of 1 to 100, how confident are you that you can correctly answer questions about what you have just learned?” The rating scale for these JOLs ranged from 0 to 100. For data analyses, JOL bias and accuracy scores were computed (Schraw, 2009). The bias score was computed by subtracting the aggregated learning outcome from JOL. It represents the discrepancy between self-assessment and actual performance, with negative scores indicating an overestimation. The JOL accuracy is computed from the absolute value of the bias score. The closer the accuracy score is to zero, the better learners assess their learning, thus the more accurate they are.

### 3.5 Procedure

The study was conducted online. Each participant was supervised one-to-one by a trained student assistant throughout the study. After instructions and compensation information, participants provided informed consent. Participants then received an anonymous code and shared their screen with the assistant to verify full-screen playback of the learning video and to prevent pausing. Then all participants completed the prior knowledge test. Next, participants worked in the learning environment and watched the software tutorial in one of the five signal redundancy conditions. Immediately afterward, participants completed the cognitive load questionnaire and provided judgments of learning and situational interest. Learning outcomes were assessed. Finally, participants reported perceived mental effort and mental load while answering the questions and provided demographic information.

## 4 Results

We set *α* = 0.05. We report *η_p_^2^* as the effect size (small, medium, and large: 0.01, 0.06, 0.14). To evaluate anticipated null effects between randomized groups in demographic data and learning prerequisites, we conducted Bayesian analyses in JASP using the JZS Cauchy prior with scale *r* = 0.707 (Morey et al., 2016). Evidence strength was interpreted using *BF_01_*. All frequentist analyses were performed in IBM SPSS Statistics (30.0), and all Bayesian analyses were performed in JASP (0.95.4).

### 4.1 Preliminary analyses

We conducted a factorial Bayesian ANOVA across all groups to assess whether randomization resulted in comparable groups. In the Bayesian ANOVAs, we found evidence supporting the null hypothesis for our demographic and prerequisite variables across groups (age: *BF_01_* = 14.80; prior knowledge: *BF_01_* = 8.12; software expertise: *BF_01_* = 17.20). The employed Bayesian contingency table showed evidence for the null hypothesis for gender (*BF_01_* = 21.03) and the highest degree (*BF_01_* = 20.43).

A Pearson correlation was conducted to examine the relationship between software expertise and prior knowledge, and to assess the relationships between these variables and the dependent variables (retention, transfer, and cognitive load). The analysis revealed a statistically significant positive correlation between software expertise and retention, *r* (99) = 0.246, *p* = 0.013, indicating that higher levels of software expertise were associated with greater retention. All other correlations between participants’ characteristics and the dependent variables were not statistically significant. We will therefore include software experience as a covariate for the respective analyses.

### 4.2 Effect of the amount of signaling on retention and transfer

**Figure 2:**
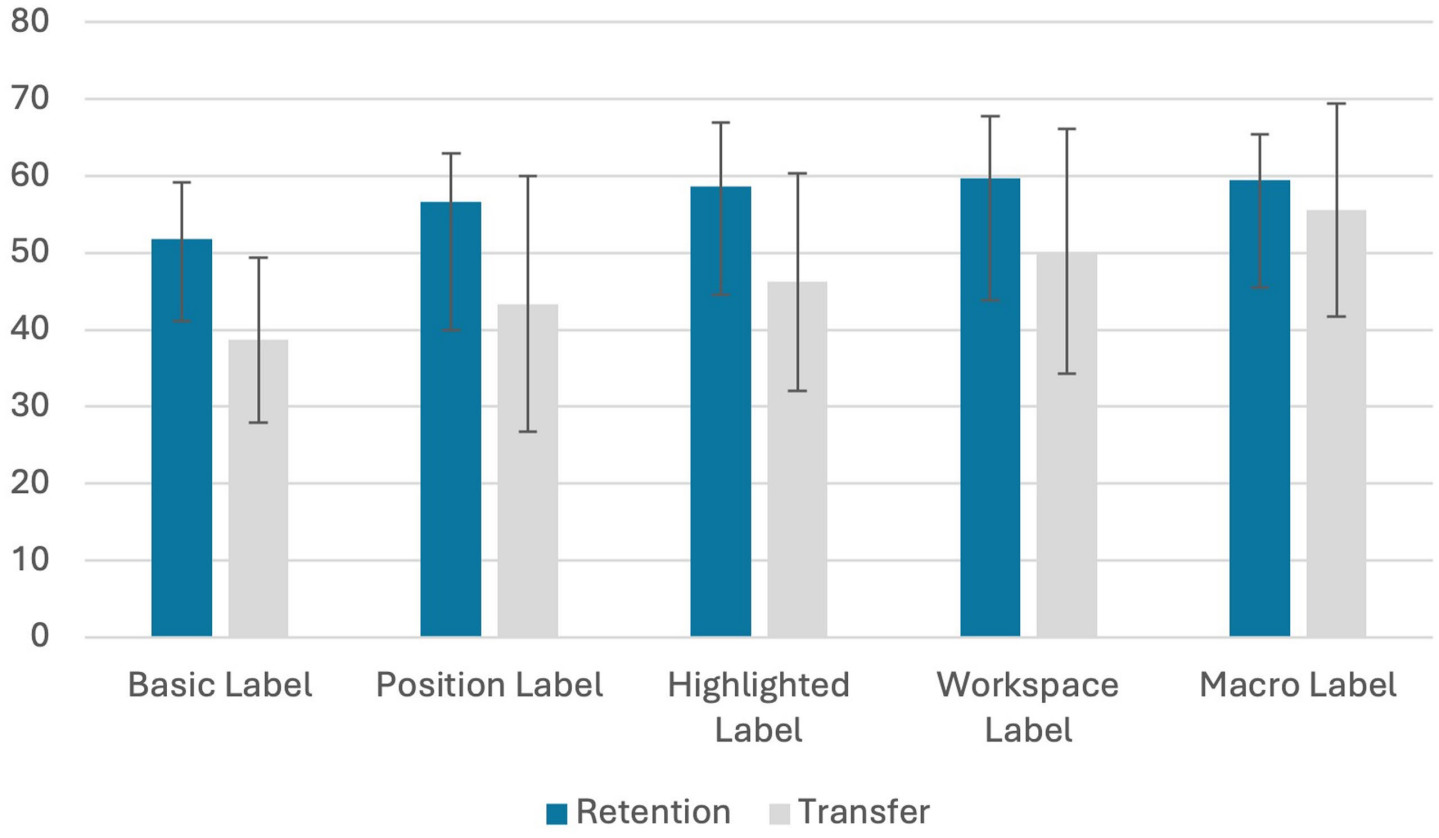
Mean learning outcomes scores and standard deviations by condition (in percent of success).

Consistent with our learning outcome hypotheses (H1), we expected that greater signaling would improve the learning outcome. A planned linear contrast tested the increase in retention and transfer as a function of the amount of signaling, evaluated across the five hierarchical groups. Software expertise was included as a covariate in the analyses. For both measures the contrast was significant; retention: *b* = 17.54, *p* < 0.001, CI^95^ [7.65, 27.43]; *F* (1,95) = 12.40, *p* < 0.001, *η_p_^2^* = 0.115; transfer: *b* = 39.09, *p* < 0.001, CI^95^ [19.64, 58.53]; *F* (1,95) = 15.93, *p* < 0.001, *η_p_^2^* = 0.144 (Figure 2). To test for the robustness of our results, we also tested the hypotheses without the covariate. The pattern of results was the similar retention: *b* = 18.23, *p* < 0.001, CI^95^ [8.13, 28.32]; transfer: *b* = 40.64, *p* < 0.001, CI^95^ [20.62, 60.66].

### 4.3 Effect of the amount of signaling on cognitive loads

**Figure 3:**
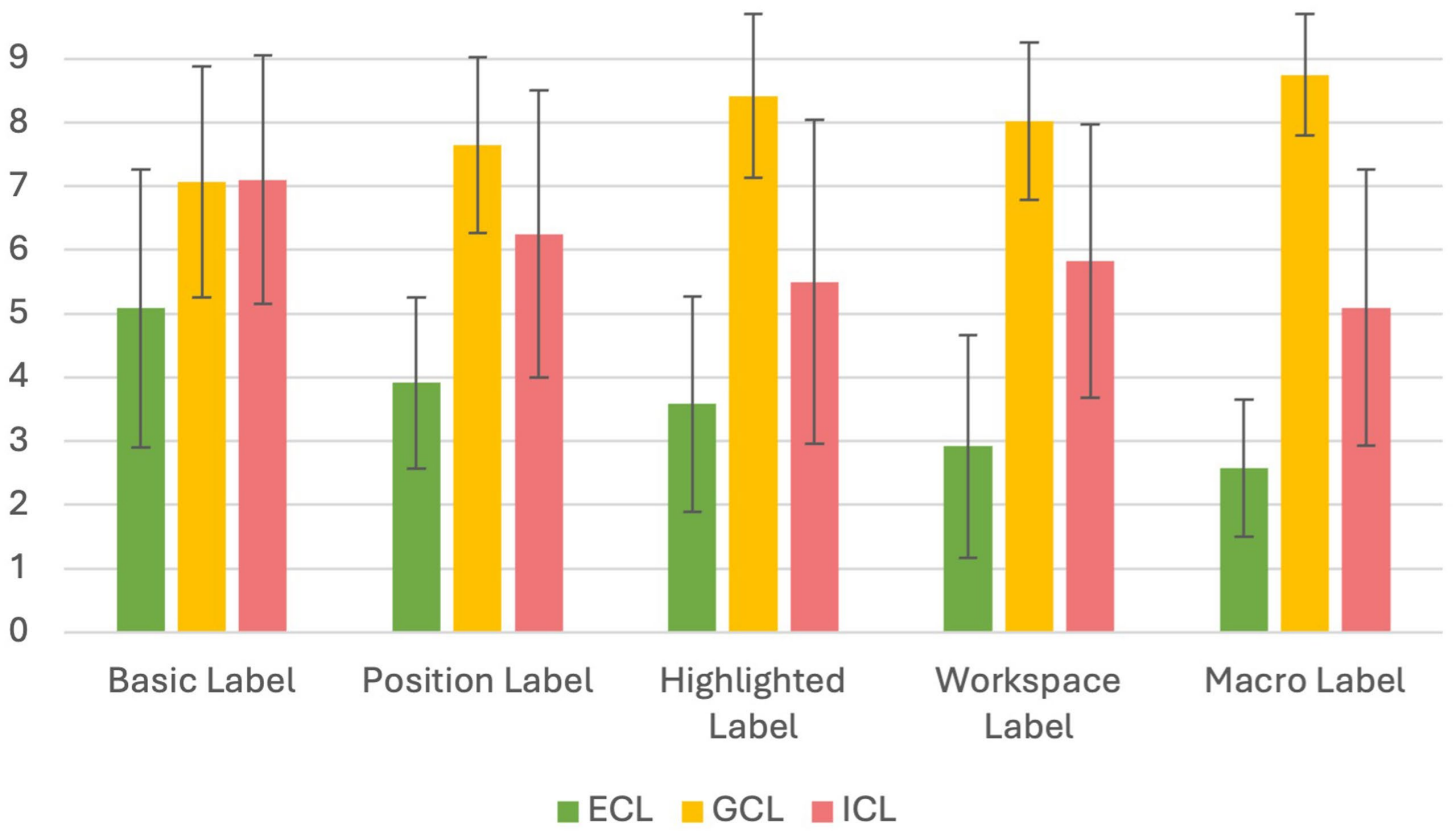
Mean score and standard deviations for measures of cognitive loads.

Following our extraneous load reduction hypothesis (H2), we expected that increasing the amount of signaling would decrease ECL. The linear contrast was significant, showing a decrease over the five groups: *b =* − 6.02, *p* < 0.001, CI^95^ [−8.31, −3.73]; *F* (1,96) = 27.28, *p* < 0.001, *η_p_^2^* = 0.221 (see Figure 3).

Following our germane load-increase hypothesis (H3), we expected that increased signaling would be associated with increased perceived GCL. The linear contrast was significant: *b* = 3.72, *p* < 0.001, CI^95^ [1.83, 5.62], *F* (1,96) = 15.29, *p* < 0.001, *η_p_^2^* = 0.137.

### 4.4 Exploratory analyses

Because supporting multimedia processing does not affect the complexity of the content to be learned, we examined whether the amount of signaling in the material influenced perceived ICL ratings. As the CLT would not predict any differences, we used a Bayesian one-way ANOVA. The Bayes factor indicated no substantial evidence for any model, *BF_01_* = 1.02. However, the planned contrast for the other load types revealed a significant linear trend: *b* = −4.43, *p* < 0.005, CI^95^ [−7.52, −1.35]. Learners perceived the learning material as less complex when more signals were provided.

We conducted the same linear contrast on mental load and mental effort while answering the questions. Mental load showed a linear decrease with greater signaling: *b* = −3.87, *p* < 0.001, CI^95^ [−5.17, −2.56]. Mental effort showed a reverse pattern: investment increased with higher signaling [*b* = 2.45, *p* < 0.001, CI95 (1.10, 3.80)].

We conducted the same linear contrast on situational interest, JOLs, and the two accuracy scores. We found no significant contrast (situational interest: *p* = 0.18, *BF_01_* = 3.56; JOLs: *p* = 0.07, *BF_01_* = 3.26; metamemory: *p* = 0.58, *BF_01_* = 12.16; metacomprehension: *p* = 0.29, *BF_01_* = 5.76).

## 5 Discussion

In the context of software video tutorials—a visually demanding format that requires fine-grained attention to interface details—increasing the redundancy of signaling (i.e., more frequent and explicit cues) yielded higher learning outcomes, with the linear trend especially pronounced for transfer. This pattern counters a classic redundancy concern and aligns with meta-analytic evidence that signaling reliably improves retention and transfer by directing attention to essential elements and reducing inefficient search processes (Alpizar et al., 2020; Schneider et al., 2018). These results therefore support our Learning Outcome Hypothesis (H1).

Consistent with our Extraneous Load Reduction Hypothesis (H2), ECL decreased linearly with increasing signaling redundancy. This conforms to Cognitive Load Theory (CLT) and multimedia learning explanations of the signaling effect: cues guide visual attention to relevant information (often synchronized with narration), thereby reducing nonessential processing and search and lowering extraneous demands (Beege et al., 2020; van Gog, 2021). Eye-tracking studies corroborate that signaling focuses fixations on task-relevant regions and improves time efficiency (Jamet, 2014; Ozcelik et al., 2009). Together, these findings support the view that signaling operates primarily through an ECL-reduction mechanism in media-rich learning contexts.

We also observed a significant linear increase in Germane Cognitive Load (GCL) with higher signaling redundancy, confirming our Germane Load Increase Hypothesis (H3). One mechanism is straightforward: reducing ECL frees working memory resources, allowing learners to invest them in schema construction and deeper processing (van Gog et al., 2010). A complementary mechanism is motivational: recent integrations of CLT with Self-Determination Theory indicate that load-reducing instructional strategies and autonomy-supportive structuring are associated with lower ECL and higher autonomous motivation and engagement, which, in turn, support deeper learning (Evans et al., 2024). In digital learning settings, even minimal choice features can enhance perceived autonomy and reduce intrinsic load, translating into better retention and transfer. This suggests the plausible following pathway: signal redundancy ➔ reduced ECL ➔ higher perceived autonomy ➔ increased ECL ➔ higher perceived autonomy ➔ increased investment (GCL). Meta-analytic syntheses also report positive effects of signaling on motivation/affect alongside reduced cognitive load, reinforcing this dual-route explanation.

Exploratory analyses revealed an unexpected linear decrease in Intrinsic Cognitive Load (ICL) with increasing signaling. Theoretically, ICL depends on the interactivity of content and learners’ prior knowledge (Endres et al., 2022). Because we did not manipulate content complexity, this pattern likely reflects a reduction in perceived complexity rather than objective complexity (Chen et al., 2023). CLT’s element interactivity account clarifies that clarifying structure and relationships (as signaling does) can make complex content easier to understand, especially for less experienced learners (Chen et al., 2023).

We further observed lower mental load and higher mental effort in the high-signaling condition. Following the distinction proposed in the MEL-TS framework, this pattern suggests that learners experienced the assessment phase as less demanding while still investing effort intentionally in task completion (Endres et al., 2025a). This is relevant because signaling may affect not only initial encoding, but also subsequent stages of processing, such as follow-up lessons or learning tasks. Future research could therefore examine signaling effects across sequences of tasks, for example, from learning multiple materials to test whether the benefits of signaling increase when multiple learning phases follow.

We further observed lower mental load and higher mental effort in the high-signaling condition. Distinguishing mental load (subjective difficulty) from cognitive load components is essential for valid interpretation, as reporting subjective effort measures keeps conclusions grounded in observed data while avoiding overreach into unmeasured constructs (Krell et al., 2022). The combined pattern (lower load, higher effort) is consistent with more efficient processing and suggests potential longer-term benefits via consolidation of more stable mental models (Schneider et al., 2018). This aligns with broader work showing that self-assessment accuracy depends strongly on the nature of cues learners use for their monitoring, especially in early skill acquisition (Waldeyer et al., 2024).

Finally, metacognitive variables—including judgments of learning, metamemory, and metacomprehension—did not differ significantly across conditions. Bayesian analyses provided substantial evidence for the null hypothesis, indicating that learners were largely unaware of the beneficial effects of increased signaling in software tutorials, consistent with prior reports that objective improvements do not necessarily translate into metacognitive insight (Schneider et al., 2018).

### 5.1 Boundary conditions, limitations, and perspectives

Although increasing signaling redundancy improved outcomes in our software video tutorial context, several boundary conditions temper generalization. First, the expertise reversal effect suggests that guidance beneficial for novices can become redundant—or even detrimental—for more knowledgeable learners; future work should therefore stratify by prior knowledge or adapt signaling dynamically (Kalyuga, 2007; Sweller et al., 2003). Second, meta-analyses show that the benefits of signaling are robust overall but that moderation by context and design features (e.g., pacing, medium, cue type) is inconsistent, implying that task characteristics and cue design matter (Alpizar et al., 2020; Schneider et al., 2018). In video-based software instruction, synchronized visual cues can preserve temporal contiguity with narration and reduce visual search costs, but these advantages depend on careful alignment of cue timing and spatial placement (Jamet, 2014; Jamet and Fernandez, 2016). Recent work also extends the signaling principle to auditory modalities. Désiron and Schneider (2024a,b) showed that prosodic cues in podcasts (e.g., variations in volume and pace) can enhance learning outcomes, particularly when combined. However, they may also increase cognitive load, leading to an underestimation of learning. This suggests that signaling is multimodal and that its cognitive and metacognitive effects may vary depending on cue characteristics and modality. Finally, although our pattern of reduced extraneous load and increased germane load aligns with CLT, motivation may also mediate these effects; load-reducing, autonomy-supportive designs tend to be associated with greater autonomous motivation and engagement, which can amplify deep processing (Evans et al., 2024; Schneider et al., 2018).

Our study has limitations that inform future research. First, we observed a decrease in Intrinsic Cognitive Load (ICL) with increased signaling despite not manipulating content complexity. Within CLT’s element interactivity framework, this likely reflects a drop in perceived complexity rather than a change in objective complexity. Future experiments should manipulate element interactivity directly (e.g., by altering the number and interdependence of interface elements) and measure both objective structure and subjective appraisals to disentangle these aspects (Chen et al., 2023). Second, our inferences about cognitive load rely on subjective ratings. While such measures are standard, a validity-aware assessment strategy that triangulates subjective reports with behavioral or physiological indices would strengthen claims (Krell et al., 2022). Third, we did not include formal measures of prior motivation (e.g., individual/topic interest); given evidence that design can jointly influence cognitive load and motivational quality, incorporating autonomous versus controlled motivation (and perceived competence/autonomy) would clarify whether signaling’s benefits are partly motivational (Evans et al., 2024; Schneider et al., 2018).

### 5.2 Practical implications for designing software tutorials

From an applied perspective, our findings support the use of systematic, frequent signaling in video tutorials, such as arrows, color-coding, and spotlighting, synchronized with narration and key interface events. These attention-guiding cues reduce ECL, enhance transfer, and can be implemented without compromising self-paced interaction or autonomy (Jamet and Fernandez, 2016; Beege et al., 2020). Designers should, however, respect coherence and avoid decorative or irrelevant cues to prevent the reintroduction of extraneous processing (van Gog, 2021). At the same time, not every visually salient addition is necessarily beneficial; recent work suggests that the effects of color-based attention guidance depend on whether visually enhanced information is learning-relevant or irrelevant, indicating that cue relevance and design need to be considered carefully (Désiron and Schneider, 2024a,b). When feasible, lightweight choice elements (topic or path) may complement signaling by supporting autonomy and reducing perceived intrinsic load (Schneider et al., 2018).

## Data Availability

The raw data supporting the conclusions of this article will be made available by the authors, without undue reservation.
